# Systematic Identification, Evolution and Expression Analysis of the *Zea mays PHT1* Gene Family Reveals Several New Members Involved in Root Colonization by Arbuscular Mycorrhizal Fungi

**DOI:** 10.3390/ijms17060930

**Published:** 2016-06-13

**Authors:** Fang Liu, Yunjian Xu, Huanhuan Jiang, Chaosheng Jiang, Yibin Du, Cheng Gong, Wei Wang, Suwen Zhu, Guomin Han, Beijiu Cheng

**Affiliations:** Key Laboratory of Crop Biology of Anhui Province, Anhui Agricultural University, No. 130, Changjiang West Road, Hefei 230036, China; weishanren163@163.com (F.L.); xuyunjian1992@163.com (Y.X.); jh13526462127@163.com (H.J.); myjiangchaosheng@163.com (C.J.); 15605659633@163.com (Y.D.); gongcheng520@126.com (C.G.); wangweisys@ahau.edu.cn (W.W.); zhusuwen@126.com (S.Z.)

**Keywords:** *Zea mays*, phosphate transporters, evolution, expression, AMF-inducible, promoter

## Abstract

The Phosphate Transporter1 (*PHT1*) family of genes plays pivotal roles in the uptake of inorganic phosphate from soils. However, there is no comprehensive report on the *PHT1* family in *Zea mays* based on the whole genome. In the present study, a total of 13 putative *PHT1* genes (*ZmPHT1;1* to *13*) were identified in the inbred line B73 genome by bioinformatics methods. Then, their function was investigated by a yeast *PHO84* mutant complementary experiment and qRT-PCR. Thirteen *ZmPHT1* genes distributed on six chromosomes (1, 2, 5, 7, 8 and 10) were divided into two paralogues (Class A and Class B). *ZmPHT1;1/ZmPHT1;9* and *ZmPHT1;9/ZmPHT1;13* are produced from recent segmental duplication events. *ZmPHT1;1/ZmPHT1;13* and *ZmPHT1;8/ZmPHT1;10* are produced from early segmental duplication events. All 13 putative *ZmPHT1s* can completely or partly complement the yeast Pi-uptake mutant, and they were obviously induced in maize under low Pi conditions, except for *ZmPHT1;1* (*p* < 0.01), indicating that the overwhelming majority of *ZmPHT1* genes can respond to a low Pi condition. *ZmPHT1;2*, *ZmPHT1;4*, *ZmPHT1;6*, *ZmPHT1;7*, *ZmPHT1;9* and *ZmPHT1;11* were up-regulated by arbuscular mycorrhizal fungi (AMF), implying that these genes might participate in mediating Pi absorption and/or transport. Analysis of the promoters revealed that the MYCS and P1BS element are widely distributed on the region of different AMF-inducible *ZmPHT1* promoters. In light of the above results, five of 13 *ZmPHT1* genes were newly-identified AMF-inducible high-affinity phosphate transporters in the maize genome. Our results will lay a foundation for better understanding the *PHT1* family evolution and the molecular mechanisms of inorganic phosphate transport under AMF inoculation.

## 1. Introduction

Although phosphorus is an essential macro-elements for plant growth and development, levels of inorganic phosphate (Pi) in different types of soils are mostly insufficient for plant growth and vegetative productivity [1]. Plants have evolved multiple strategies to increase and assimilate available Pi from soil, including secretion of organic acids and phosphatases to solubilize Pi trapped in complexes, increasing the root-soil interface, forming a symbiotic association with arbuscular mycorrhizal fungi (AMF), *etc.* [1,2]. The symbiosis of plant roots with AMF is one of the important strategies. It is estimated that AMF form mycorrhizal with the roots of 80% of all terrestrial plant species and promote the growth of the host plant mainly by enhancing the phosphorus uptake [3]. The symbiosis with AMF is an important nutrition uptake method, which can contribute to up to 90% of plant phosphorus requirements [4,5].

Plants have two types of Pi uptake systems, *i.e.*, a high-affinity Pi uptake system and a low-affinity Pi uptake system. Phosphate Transporter1 (PHT1) are high-affinity Pi transporters, which play pivotal roles in the uptake of phosphorus from soils under phosphorus-limited conditions [1]. Furthermore, a previous study suggested that dual affinity PHT1 transporters likely exist in *Arabidopsis* [6]. The majority of *PHT1* genes are mainly expressed in root epidermal cells and outer cortex, implying their potential involvement in phosphate uptake from the soil [2]. Besides the direct uptake of phosphorus from soils around the roots, plants often acquire additional phosphorus from deep soils with the help of AMF fungal hyphae. During plant-AMF association, three types of *PHT1* genes are involved in the absorption, *i.e.*, fungal *PHT1* genes, plant *PHT1* genes and plant *PHT1* genes induced by AMF [2]. The fungi first assimilate phosphorus by fungal PHT1 transporters and then transfer phosphorus to the plant via AMF-inducible plant PHT1 transporters [2,7].

Members of the plant *PHT1* gene family share homologies with the yeast PHO84 Pi transporter and belong to the phosphate-H^+^ symporter family [8]. They have a similar structure, *i.e.*, 12 putative transmembrane (TM) domains, hydrophilic N- and C-terminals and a hydrophilic loop between TM6 and TM7 [8]. The first crystal structure of high-affinity phosphate transporter (PiPT) from *Piriformospora indica* was solved recently. The structure shows exit pathways of protons and phosphate and suggests a phosphate transport mechanism called “rocker-switch” [9]. Several members of the *PHT1* family in dicot and monocot plants, particularly from many economically-important plant species, have been identified [1,2]. The first plant *PHT1* gene was isolated from *Arabidopsis thaliana* and exhibits similarities to *PHO84* [10]. A large number of plant genomes have been sequenced with next generation sequencing technology [11,12,13]. All of the members of the *PHT1* family have been identified in some species’ whole genome. For example, 9 members of the *PHT1* family were identified in the *Arabidopsis* genome [14], 13 in *Oryza sativa* [15], 8 in *Solanum lycopersicum* [16], 10 in *Solanum tuberosum* [16], 6 in *Astragalus sinicus* [17], 14 in *Glycine max* [18], 13 in *Brachypodium distachyon* [19], 11 in *Sorghum bicolor* [17], 12 in *Setaria italic* [20], *etc.* A limited number of AMF-inducible Pi transporters in plants have been identified in *Medicago truncatula* [21], *Astragalus sinicus* [17], *Oryza sativa* (*O. sativa*) [15,22], *Solanum tuberosum* (*S. tuberosum*) [23], *Solanum lycopersicum* [24], *Triticum aestivum* [25], *Brachypodium distachyon* (*B. distachyon*) [19], *Setaria italic* (*S*. *italic*) [20] and *Zea mays* (*Z. mays*) [26].

As one of the most important crops in the world, maize is grown widely, but multiple environmental stresses limit its production, especially by different degrees of low Pi stress. Five genes encoding PHT1 phosphate transporters were identified from a commercial hybrid Maize cDNA library [26]; the expression level of *ZEAma:PHT1;6* was much higher in plants colonized by AMF than that in non-mycorrhizal maize roots [27]. Compared to the investigation on *PHT1* genes in whole genome sequenced plants, such as *B. distachyon*, *S. bicolor* and *O. sativa*, there is no comprehensive report on *PHT1* family genes in the *Z. mays* whole genome level, particularly in inbred lines.

In this study, members of PHT1 family genes in *Z. mays* elite inbred line B73 genome were identified by bioinformatics and experimental investigations, and the characteristics of the genes were also investigated under a phosphorus-adverse environment, as well as the AMF inoculation condition. The detailed identification and functional analyses of *Z. mays* *PHT1* genes might help us to find out some new approaches to improve the phosphate uptake efficiency of maize.

## 2. Results

### 2.1. Identification and Properties of Putative Z. mays PHT1 Genes

a total of 13 putative genes were identified in maize inbred line B73 genome and were denominated as *ZmPHT1;1* to *ZmPHT1;13* (Table 1). All 13 ZmPHT1 proteins contain a highly conserved Sugar_tr domain (PF00083) and encode proteins varying from 509 to 597 AA. The minimum and maximum theoretical molecular weights are 56.22 kDa (*ZmPHT1;5*) and 66.07 kDa (*ZmPHT1;12*), respectively. The amino acid number and molecular weight of these proteins have slight differences. Besides, all 13 genes are alkaline, with the isoelectric point ranging from 7.58 to 9.41. All ZmPHT1 proteins contain 12 putative membrane-spanning domains, as shown in Figure S1.

### 2.2. Phylogenetic Analysis of PHT1 Transporters in Z. mays

**Figure 1:**
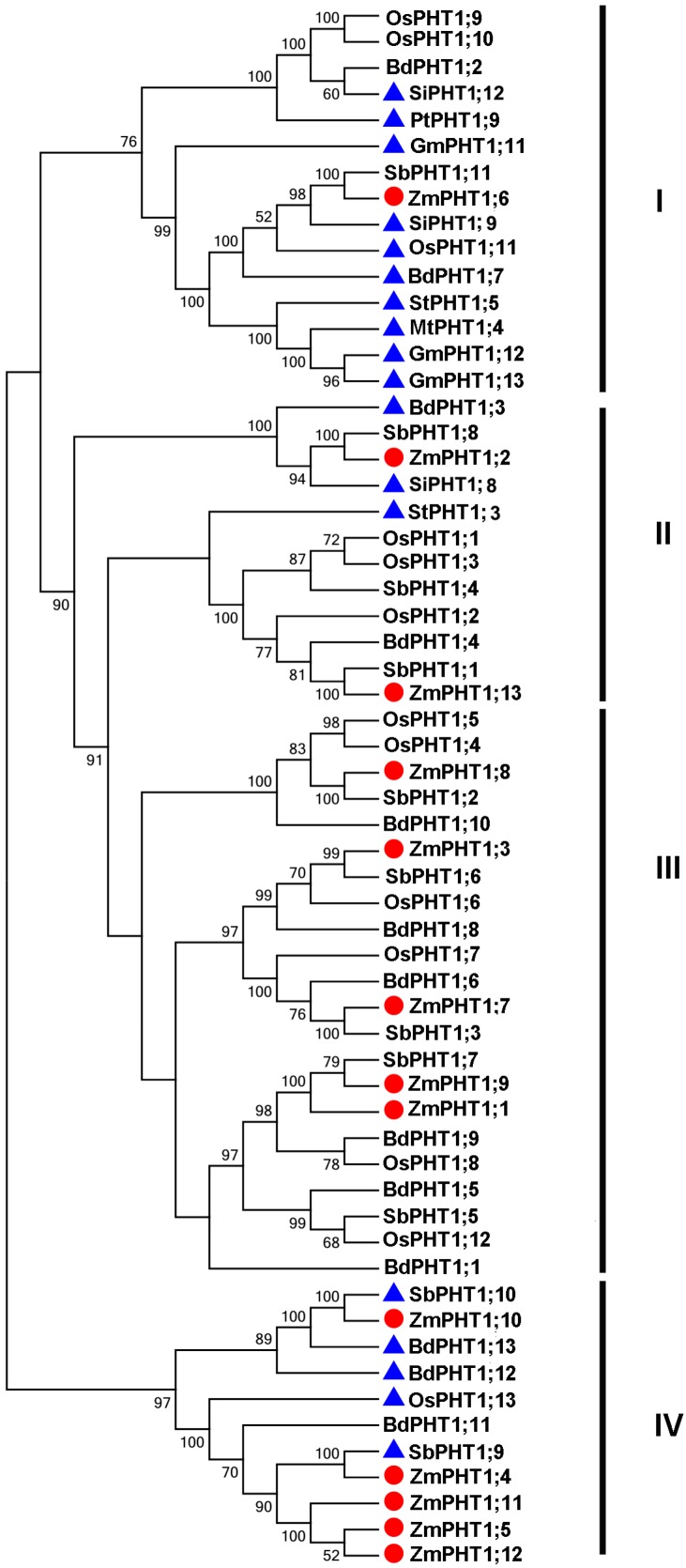
Phylogenetic analysis of PHT1 transporters between maize and other plants. Roman numerals (**I**–**IV**) indicate the four *PHT1* subfamilies. Transporters from maize (ZmPHT1;1 to ZmPHT1;13), rice (OsPHT1;1 to OsPHT1;13), sorghum (SbPHT1;1 to SbPHT1;11), Brachypodium (BdPHT1;1 to BdPHT1;13) and some recently-identified AM-inducible PHT1 transporters [20]. ZmPHT1s are indicated by filled red circles, and AMF-inducible PHT1s in other plants are indicated by filled blue triangles.

A phylogenetic tree of PHT1 transporters was constructed by multiple sequence alignment of proteins from maize, rice, sorghum Brachypodium and AM-induced PHT1 transporters from *G. max*, *Medicago truncatula*, *P. trichocarpa*, *S. italic* and *S. tuberosum* [20] (Figure 1). ZmPHT1 proteins were divided into four subfamilies, Classes I to IV. Class I was monocot and dicot AM-inducible PHT1s, only containing ZmPHT1;6. ZmPHT1;1, ZmPHT1;3, ZmPHT1;7 and ZmPHT1;9 are involved in monocot Class III without AM-inducible PHT1s. ZmPHT1;4, ZmPHT1;5, ZmPHT1;8, ZmPHT1;10, ZmPHT1;11 and ZmPHT1;12 together with other monocot AM-inducible proteins fall into Class IV. ZmPHT1;2 and ZmPHT1;13 are involved in Class II.

### 2.3. The Conserved Structure Analysis of Maize PHT1 Proteins

**Figure 2:**
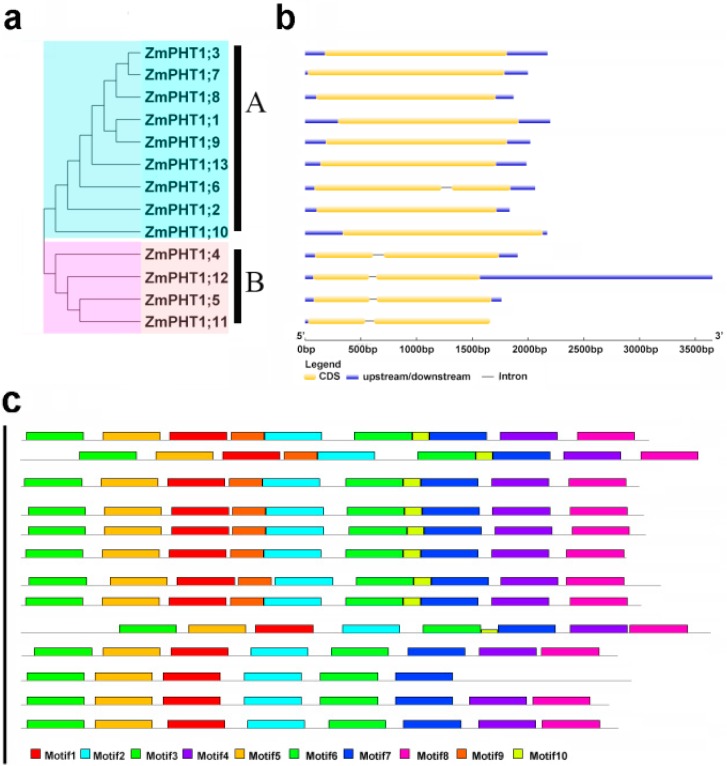
Phylogenetic tree, motif and gene structure of *ZmPHT1* genes. (**a**) The phylogenetic tree contains only ZmPHT1s, which were divided into two groups (Class A and Class B); (**b**) exons are indicated by grey boxes, and introns are indicated by single lines; black lines represent untranslated regions (UTR); (**c**) motifs’ analysis; different colors represent different motifs.

The phylogenetic tree of 13 ZmPHT1 transporters was divided into two subfamilies (Subfamily A and Subfamily B) (Figure 2). Ten conserved motifs were identified in ZmPHT1 proteins by MEME software. Proteins in Subfamily A contained two specific motifs (motifs 9 and 10) compared to Subfamily B, whereas the functions of the two motifs are unknown. Besides, motifs 1, 2, 4, 5 encoded Sugar_tr domains and motifs 3, 7, 8 encoded transmembrane domains (Figure S1). Based on the hydropathy plots in the homology modeling analysis, these genes commonly encode integral membrane proteins containing 12 hydrophilic loops between TM6 and TM7, forming a 6 + 6 configuration.

### 2.4. Distribution and Genomic Duplications of Maize PHT1 Genes

**Figure 3:**
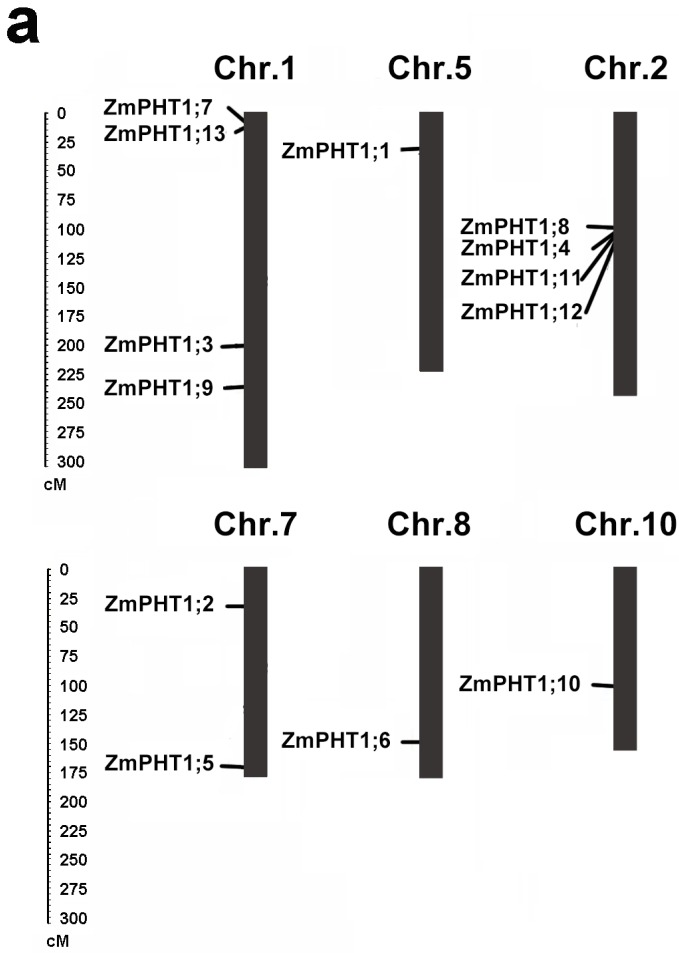

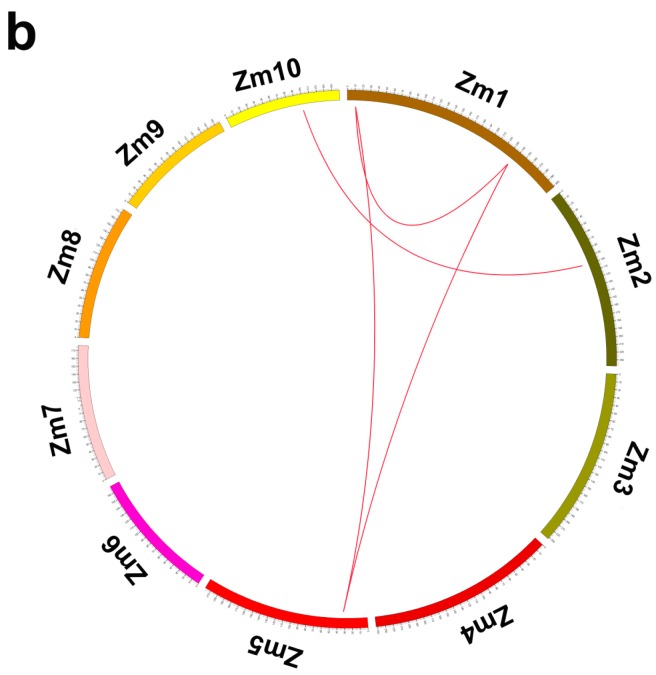
Distribution and synteny of *ZmPHT1* genes. (**a**) Distribution of *ZmPHT1* genes on the maize chromosomes. On the top of each bar is the chromosome number; (**b**) synteny of *ZmPHT1* genes. Zm1–10 represented maize chromosome 1–10, indicated by colored boxes. Chromosome box numbers represent sequence lengths in megabases. All of the syntenic genes were located in the map and linked by red lines.

Compared to the whole maize genome, thirteen putative *PHT1* genes are located on chromosomes 1, 2, 5, 7 and 10, respectively (Figure 3A). Four duplicated pairs of *ZmPHT1* genes (*ZmPHT1;1*/*ZmPHT1;9*, *ZmPHT1;9*/*ZmPHT1;13*, *ZmPHT1;1*/*ZmPHT1;13* and *ZmPHT1;8*/*ZmPHT1;10*) were identified on the synteny map (Figure 3B) and were a segmental duplication event. All of their Ka/Ks values (Table 2, Figure S2) were <1.0, except *ZmPHT1;9*/*ZmPHT1;13* (2.76). Statistical analysis indicated that *ZmPHT1;1*/*ZmPHT1;9*, *ZmPHT1;9*/*ZmPHT1;13*, *ZmPHT1;1*/*ZmPHT1;13* and *ZmPHT1;8*/*ZmPHT1;10* were produced approximately 12.36, 7.91, 32.72 and 42.05 Mya (millions of years ago), respectively (Table 2). Further analysis revealed that amino acid sequence similarity between ZmPHT1;1/ZmPHT1;9, ZmPHT1;9/ZmPHT1;13, ZmPHT1;1/ZmPHT1;13 and ZmPHT1;8/ZmPHT1;10 was high (93.9%, 73.38%, 73.33% and 50.72%, respectively).

### 2.5. Tissue Specificity of ZmPHT1 Gene Expressions

**Figure 4:**
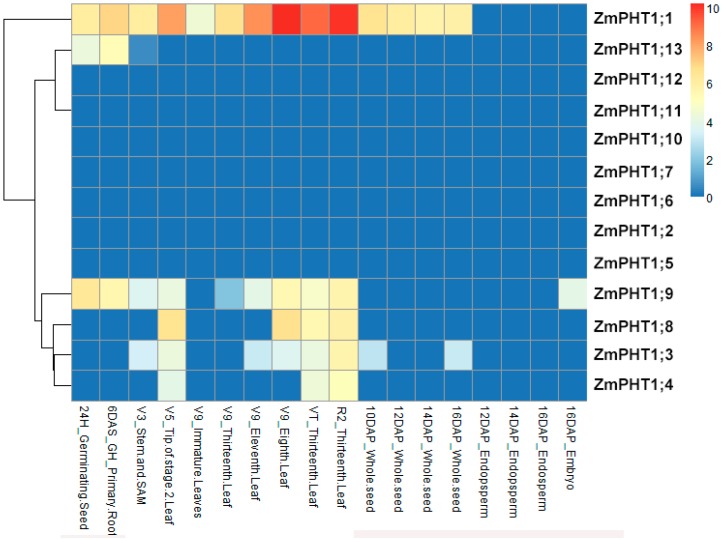
Heat map of maize *ZmPHT1* genes. The expression level of each *ZmPHT1* genes can be estimated based on the scale to the right. Red indicates high expression level; yellow indicates medium expression level; and blue indicates low expression level. H, hours; DAS, days after sowing; GH, greenhouse; V, vegetative; DAP, days after pollination; VT, vegetative tasseling; R, reproductive.

*ZmPHT1* expression patterns were analyzed by HeapMap (Figure 4) [28]. The result showed that seven genes (*ZmPHT1;2*, *ZmPHT1;5*, *ZmPHT1;6*, *ZmPHT1;7*, *ZmPHT1;10*, *ZmPHT1;11* and *ZmPHT1;12*) had no expression in any tissues, and *ZmPHT1;1*, *ZmPHT1;3*, *ZmPHT1;4*, *ZmPHT1;8* and *ZmPHT1;9* were expressed mainly in root and leaf, indicating that they play a significant role in Pi uptake and redistribution.

### 2.6. Analysis of the Inorganic Phosphate (Pi) Transport Ability of ZmPHT1 Genes in a Pi-Uptake-Defective Yeast Strain

**Figure 5:**
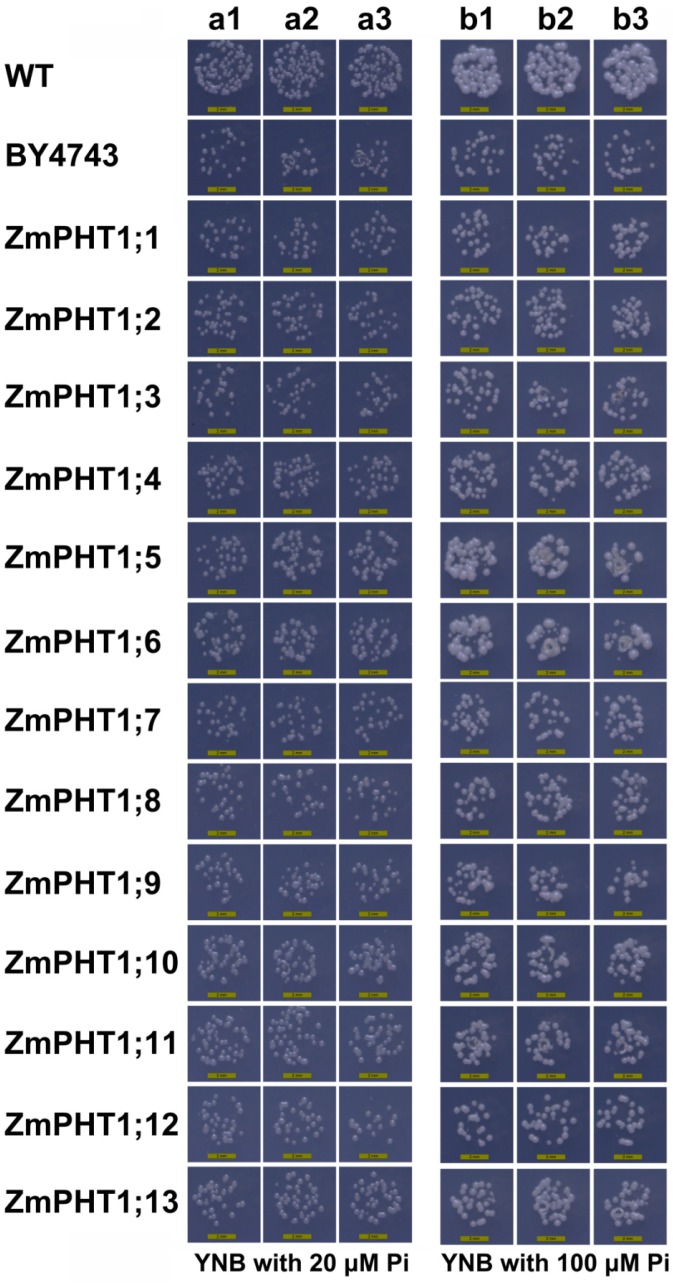
Functional characterizations of *ZmPHT1s* in a yeast inorganic phosphate (Pi) transport mutant. Under two Pi conditions three repeat experiments were set respectively. Repeat experiments were represented by a1, a2, a3 under 20 µM and b1, b2, b3 under 100 µM Pi conditions.

To analyze the Pi transport characteristics of 13 ZmPHT1 transporters, the whole open reading frames (ORF) were cloned into a yeast expression vector separately (pYES2). Each of these constructs was transformed into the yeast Pi transport mutant (BY4743) lacking the high-affinity Pi transporter gene *PHO84*. Transformed yeasts were cultured in YNB (Yeast Nitrogen Base) with low Pi condition (20 µM and 100 µM Pi). Results showed that the mutant cells of the yeast BY4743 strain grow poorly under low Pi conditions, while cells of *ZmPHT1s* transformants grow more or larger than that of the control, especially *ZmPHT1;5*, *ZmPHT1;6*, *ZmPHT1;10*, *ZmPHT1;11* and *ZmPHT1;13* (Figure 5), suggesting that all 13 putative ZmPHT1 proteins can completely or partly complement the yeast Pi transport mutant under low Pi statues.

### 2.7. Influence of Pi Availability and AM Symbiosis on ZmPHT1 Gene Expression

**Figure 6:**
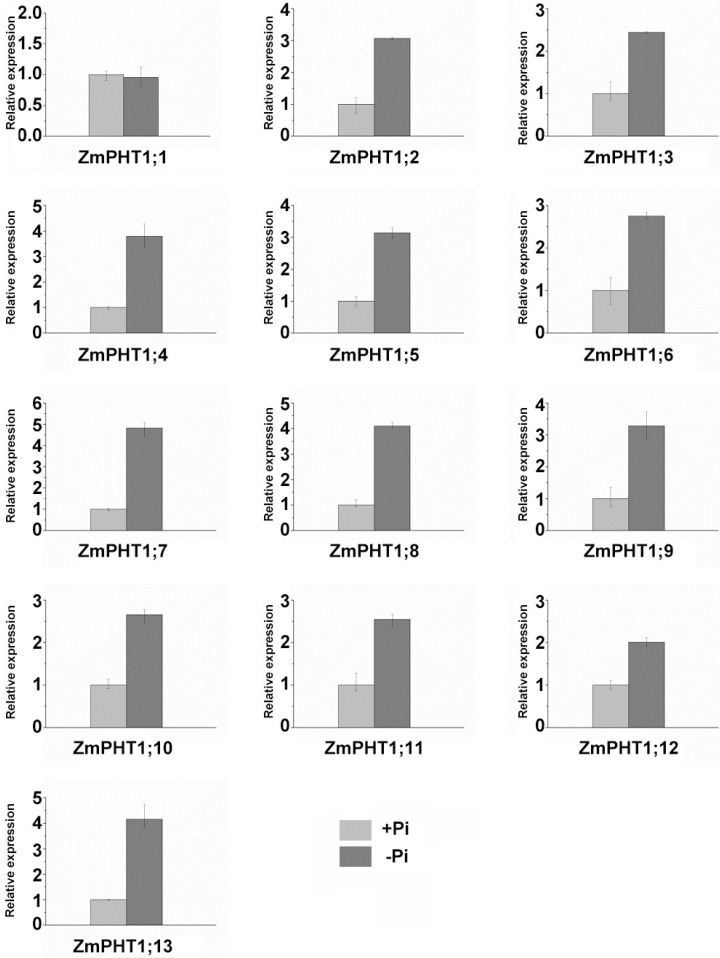
Expression pattern analyses of *ZmPHT1* genes in root related to Pi availability. Maize was grown in nutrient solution containing 50 µM Pi and 5 mM Pi and sampled 40 days after treatment initiation. Data points are the means ± SD (*n* = 3 biological replicates of three plants each).

Transcripts levels of *ZmPHT1* genes responding to Pi concentration were investigated using qRT-PCR under conditions of high Pi and low Pi (Figure 6). All of the *ZmPHT1* genes were significantly induced under the low Pi condition except *ZmPHT1;1* (*p* < 0.01), indicating that twelve of 13 *ZmPHT1* genes can respond to low Pi.

**Figure 7:**
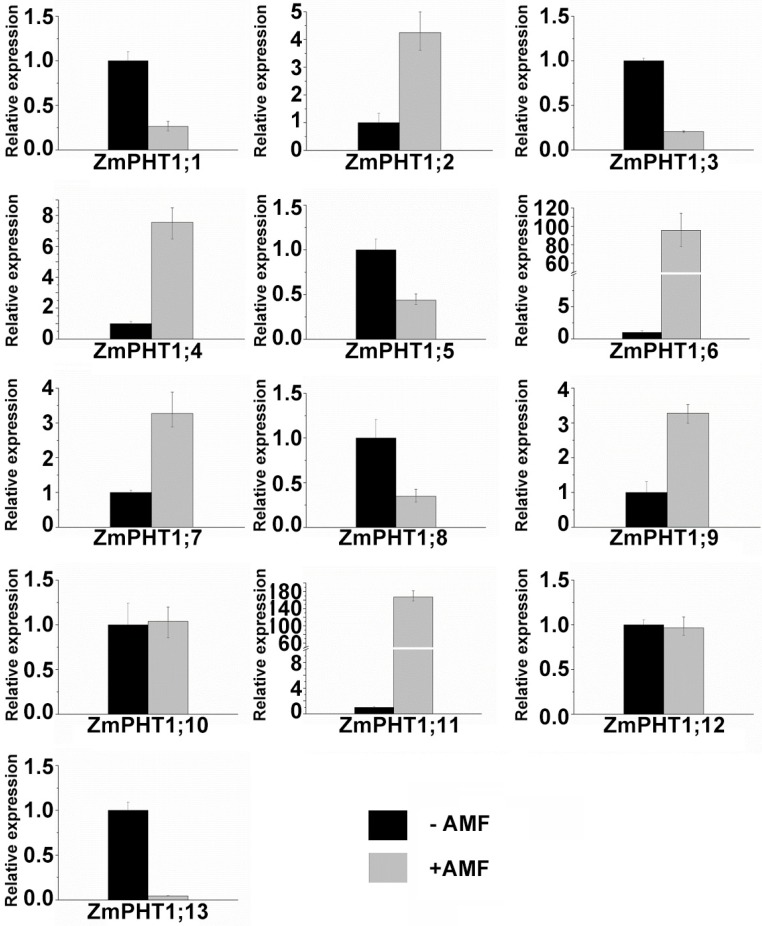
qRT-PCR analysis of *ZmPHT1* genes in root samples in response to AMF inoculation under low Pi supply. Maize were grown in a low Pi (50 µM) condition and an inoculation with autoclaved and active *Glomus etunicatum*, respectively. Data points are the means ± SD (*n* = 3 biological replicates of three plants each).

The expression of ZmPHT1 transporters in response to mycorrhizal colonization and non-inoculated roots was also assessed (Figure 7). *ZmPHT1;2*, *ZmPHT1;4*, *ZmPHT1;6* and *ZmPHT1;11* in the classes (I, II and IV) harboring AMF-inducible PHT1 transporters were induced in the AM fungal symbiont, except *ZmPHT1;5* and *ZmPHT1;8*. In Class III, *ZmPHT1;1*, *ZmPHT1;3* and *ZmPHT1;13* were downregulated in inoculated profiles, whereas *ZmPHT1;7* and *ZmPHT1;9* were significantly upregulated (>3-fold). Conspicuously, the relative expression of the *ZmPHT1;6* had a much higher transcript level in AM-induced roots than non-inoculated roots.

### 2.8. Identification of the Regulatory Cis-Elements

**Figure 8:**
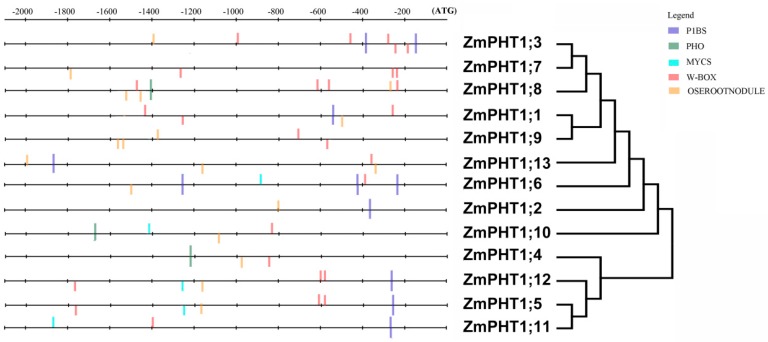
Analysis of regulatory elements in the promoters of *ZmPHT1* genes. Three previously reported Pi-responsive motifs (P1BS, PHO and W-box) and two AM-activated motif (MYCS and OSEROOTNODULE) were searched by DNA-pattern matching arithmetic. PHO (CACGTG), P1BS (GNATATNC), W-BOX (TTGACY), MYCS (TTTCTTGTTCT, or with one nucleotide variation), OSEROOTNODULE (AAAGAT).

Regulatory elements in promoters of each *ZmPHT1* were further analyzed. Elements involved in Pi starvation and AMF induction could be detected in 2.1-kb upstream regions of *PHT1* genes (Figure 8). The number of AM-responsive elements (MYCS) and two Pi-regulated (P1BS and W-box) elements were differently distributed in the promoter regions of the 13 genes.

P1BS (GNATATNC), playing a key role in Pi starvation response [29], exists in eight *ZmPHT1s*. It could be seen that one copy of P1BS was detected in the promoter of *ZmPHT1;1*, *ZmPHT1;2*, *ZmPHT1;5*, *ZmPHT1;11*, *ZmPHT1;12* and *ZmPHT1;13*, while two copies in *ZmPHT1;*3 and three copies in *ZmPHT1;*6. No PIBS element was found in the promoter of *ZmPHT1;4*, *ZmPHT1;7*, *ZmPHT1;8*, *ZmPHT1;9* and *ZmPHT1;10.* All of the 13 *ZmPHT1s* harbored another motif, OSEROOTNODULE (AAAGAT), which was a conserved AMF-inducible element. The MYCS (TTCTTGTTC) motif is only present in putative promoter regions of the five *ZmPHT1s* (*ZmPHT1;5*, *ZmPHT1;6*, *ZmPHT1;10*, *ZmPHT1;11* and *ZmPHT1;12*); most of them could be upregulated by AM fungi (Figure 6) [29]. However, no MYCS (TTCTTGTTC) motif was detected in *ZmPHT1;2* and *ZmPHT1;4*, implying that new AM-responsive elements should exist in the promoter of the two genes.

## 3. Discussion

### 3.1. Identification of ZmPHT1 Genes

In recent studies, many *PHT1* genes have been identified from the genomes of several plants [15,16,17,18]. Although maize is an important crop species, only five high-affinity Pi transporters were isolated from the cDNA libraries of a commercial hybrid [18]. As one of the widely used and investigated elite maize inbred lines, B73, whose genome has been sequenced, however, no systematic work was reported on *PHT1* family genes in the maize genome. In our study, a total of 13 *PHT1* genes were predicted and identified from the B73 maize genome. The sequences of two *PHT1* genes from the commercial hybrid were identical to the two corresponding sequences in inbred line B73 (Figure S3, Table S4). Of the 13 maize B73 *PHT1* genes, eight of them were newly identified in this study. Similar to the *PHT1* family genes in other species, proteins of the maize *PHT1* family exhibited a high degree of identity and similar hydrophobic domains. The protein properties of putative *ZmPHT1s*, e.g., numbers of amino acid, theoretical molecular weight, putative membrane-spanning domain, *etc.*, are also similar to that of other plants [10,15,16,17,18].

Yeast mutants losing the function in Pi transporters are used widely among three systems to characterize plant Pi transporters’ function (complementation of yeast mutants defective in Pi transports, monitoring Pi uptake using *Xenopus laevis oocytes* and ectopic expression of Pi transporters in plant suspension cells) [18,30]. In the present study, the yeast Pi transport mutant BY4743, which lacks the high-affinity Pi transport gene *PHO84,* was used to verify the complementary of Pi transport abilities of each putative *ZmPHT1s*. Our results indicated that all of 13 tested *ZmPHT1s* could completely or partly complement the yeast Pi-uptake mutant under a low Pi condition, implying that all of the putative *ZmPHT1s* functions have the function of Pi uptake and transportation under a low Pi condition [18].

### 3.2. Evolutionary Expansion of ZmPHT1 Genes

Phylogenetic analysis of ZmPHT1s compared to other Pi transporters from rice, sorghum and Brachypodium indicated a similar pattern of evolutionary divergence, reflecting different biochemical and functional characters among different types of PHTs. *ZmPHT1;4*, *ZmPHT1;5*, *ZmPHT1;6*, *ZmPHT1;11* and *ZmPHT1;12* each has an intron, implying that they had arisen from a duplication event of a primordial gene [31]. Interestingly, all of the *ZmPHT1s* have no intron or only one intron in the coding regions, meaning that Pht1 genes are some of the fast responses genes related to abiotic or biotic conditions [32]. Ka/Ks values for *ZmPHT1;1*/*ZmPHT1;9*, *ZmPHT1;1*/*ZmPHT1;13* and *ZmPHT1;8*/*ZmPHT1;10* were <1.0, which indicated that the paralog *ZmPHT1* gene pairs were undergoing purifying selection in the maize evolution and, thus, may have been subfunctionalized [31]. *ZmPHT1;1*/*ZmPHT1;9* and *ZmPHT1;9*/*ZmPHT1;13* were produced in a recent duplication event, while *ZmPHT1;1*/*ZmPHT1;13* and *ZmPHT1;8*/*ZmPHT1;10* were generated in an early duplication event, corresponding to the timing of a recent and early WGD (whole genome duplication) event of maize, which occurred 12 Mya and 70 Mya, respectively [33].

### 3.3. ZmPHT1s Response to Pi Availability and AMF Inoculation

Expression of *ZmPHT1s* under different tissues showed that *ZmPHT1;1*, *ZmPHT1;3*, *ZmPHT1;4*, *ZmPHT1;8* and *ZmPHT1;9* are expressed mainly in root and leaf, suggesting that these genes should not only be involved in Pi uptake from soil, but also in the relocation of Pi across different plant tissues. Nevertheless, none of the rest *ZmPHT1s* were expressed in any tissues (Figure 4). The specialized expressions of these genes reflect well the divergence of regulatory elements requiring for formation of AM symbiosis and controlling Pi uptake. *PHT1* genes were investigated in several different plants and revealed a dominant expression in root of many *PHT1* genes, suggesting that these genes have a potential role in Pi capture and uptake [18,34].

We tested the responses of 13 *ZmPHT1s* to Pi starvation by qRT-PCR further and observed that 12 *ZmPHT1s* were upregulated under low Pi conditions, whereas only *ZmPHT1;1* was inhibited. Although the coding sequences of *ZmPHT1;1* and *ZmPHT1;9* are identical, the expression levels of two members were obviously different whether in the arbuscular mycorrhizal or low Pi supply condition. Such a discrepancy between them may be caused by the different numbers and distributions of promoter elements, such as P1BS (*ZmPHT1;1* contained one, but *ZmPHT1;9* did not) and OSEROOTNODULE (*ZmPHT1;1* contained one, but *ZmPHT1;9* contained three).

Compared to the expression patterns between AMF colonization and low Pi condition, *ZmPHT1;3*, *ZmPHT1;5*, *ZmPHT1;8* and *ZmPHT1;13* were enhanced in response to limited soil Pi availability without AMF, but downregulated in response to AMF. All of them displayed a typical characteristic of PHT1 proteins involved in the direct Pi uptake pathway of plants [1,2,23]. *ZmPHT1;2*, *ZmPHT1;4*, *ZmPHT1;6*, *ZmPHT1;7*, *ZmPHT1;9* and *ZmPHT1;11* were highly expressed in roots colonized by AMF, indicating that they are possibly involved in Pi uptake through a symbiotic pathway at the mycorrhizal interface [35]. *ZmPHT1;2*, *ZmPHT1;4*, *ZmPHT1;6* and *ZmPHT1;11*, respectively clustering in a specific clade of AM-inducible Pi transporters from other plants, were induced by mycorrhizal and non-mycorrhizal roots under low Pi conditions, proposing the direct Pi uptake pathway shift to the mycorrhizal pathway possibly during the AM symbiosis establishment.

### 3.4. Regulatory Cis-Elements in Promoters of AMF-Inducible ZmPHT1s

A phylogenetic dendrogram containing mycorrhizal-specific genes between different species was constructed. With well-supported bootstrap values, ZmPHT1;2 clustered with *Brachypodium mycorrhizal*-specific BdPHT1;3 and *S. italic* mycorrhizal-specific SiPHT1;8, whereas ZmPHT1;4, ZmPHT1;5, ZmPHT1;11 and ZmPHT1;12 clustered with rice mycorrhizal-specific OsPHT1;13 and sorghum mycorrhizal-specific SbPHT1;9 and ZmPHT1;10 clustered with AM-inducible SbPHT1;10, BdPHT1;12 and BdPHT1;13, suggesting that these genes may be involved in AM response. Interestingly, ZmPHT1;6 clustered with AM-inducible PHTs in monocot (SiPHT1;9, OsPHT1;11 and BdPHT1;7) and dicot (StPHT1;5, MtPHT1;4, GmPHT1;12 and GmPHT1;13), indicating a specific AM-inducible PHT1 clade. Indeed, several plant species, such as *Lycopersicon esculentum*, *O. sativa* and *S. tuberosum*, possess multiple AM-induced *PHT1* genes, which possibly result in a functional redundancy of Pi transport in AM symbiosis [22].

The expression pattern induced by AMF and the phylogenetic relationship of *ZmPHT1s* in this study are similar to the *PHT1* genes of other plants [2,7,15,19]. However, *ZmPHT1;5*, *ZmPHT1;8* and *ZmPHT1;12* were repressed, indicating that the influence of AMF on *ZmPHT1;s* was not only enhanced, but also repressed, and *ZmPHT1;5*, *ZmPHT1;8* or *ZmPHT1;12* should be redundantly regulated for Pi uptake in maize AM symbiosis. In Class A, *ZmPHT1;1*, *ZmPHT1;3* and *ZmPHT1;13* with more than two P1BS elements were repressed in inoculated profiles, whereas *ZmPHT1;7* (>3-fold) and *ZmPHT1;9* (>3-fold) with no P1BS element were significantly induced, suggesting that P1BS possibly has some negative influence on the expression of the AM-induced gene. However, the relative expression of the *ZmPHT1;6* genes with three P1BS, but one MYCS element, had much higher expression levels (~100-fold) in the AM-induced root, revealing that MYCS may be the main regulation factor for AM-induced gene expression compared to P1BS. It is interesting that some members of *ZmPHT1s* without the MYCS element, such as *ZmPHT1;2*, *ZmPHT1;4*, *ZmPHT1;7* and *ZmPHT1;9*, still upregulated the expression, implying that new regulation element(s) might exist in the promoter regions of some maize *PHT1* genes. Then, as a previous study had found that the CTTC motif is necessary and sufficient for plants responding to fungal colonization under Pi-limited conditions, a further study of CTTC motifs on *ZmPHT1s* was investigated [20,36]. The result showed that *ZmPHT1;4*, *ZmPHT1;7* and *ZmPHT1;9* harbored CTTC motifs in the putative promoter regions (Figure S4), which might be related to their upregulation in inoculated profiles. However, there is no such motif in the promoter of *ZmPHT1;2* (Figure S4), despite its induction following AMF colonization. Thus, the elements in the *ZmPHT1;2* promoter responsible for AMF induction remain unclear.

## 4. Materials and Methods

### 4.1. Bioinformatics Analyses

Previously reported maize, *A. thaliana* and *O. sativa* phosphate transporter proteins were used as queries against the maize genome database. After removal of overlapping sequences, the remaining genes were verified in the Pfam database (http://pfam.xfam.org/). The protein sequences were aligned by the ClustalX (Version 1.81) with default parameters. Then, the phylogenetic tree was constructed with the neighbor-joining method within MEGA 6, and the bootstrap was 1000 replicates in the mode of pairwise gap deletion [37]. The exon and intron structures of each *ZmPHT1* gene were analyzed by GSDS (Gene Structure Display Server; http://gsds.cbi.pku.edu.cn/) through alignment of their CDS with corresponding genomic DNA sequences. Conserved motifs among the *ZmPHT1* genes were examined via MEME software (Multiple Expectation Maximization for Motif Elicitation) [38], and the parameters were set as follows: the number of repetitions was choose any; the maximum number of motifs was 10; and the optimum motif width was set between 6 and 50 residues. Moreover, motif annotation was performed by the Pfam and SMART (http://smart.embl-heidelberg.de/) tools. Chromosome location was performed using the Map Inspect software [39]. The duplication pattern for each *ZmPHT1* gene was analyzed by MCScanX software [40], and the synthesis map was drawn by Circos software (http://circos.ca/) [41]. Gene clusters that met the following specific clustering criteria were selected, *i.e.*, genes arising from tandem duplication events on the same chromosome region, two or more homologous genes within a 200-kb region [42] and fragments derived from replication events [43]. The calculation of Ka, Ks was by DnaSPv5.0 [44]. The nonsynonymous substitution per synonymous site (Ka/Ks) ratio analysis was performed by sliding window analysis with the parameters as follows: window size, 150 bp; step size, 9 bp [42]. The gene expression data from Maize B73 transcriptomes were used to draw a heat map [28], including information from germinating seeds, primary roots, stems, SAMs (shoot apical meristem), leaves, endosperm, embryos and whole seeds. Promoters were analyzed by RSAT (http://floresta.eead.csic.es/rsat/) [45].

### 4.2. Plant Material and Cultivation Conditions

Maize B73 seeds germinated in a sterile environment with a photoperiod of 16 h light and 8 h dark at 28 °C. After germination, some seedlings were transferred to pots filled with sand-based culture for inoculation with AMF. Additionally, the rest of the seedlings were transferred to pots filled with sand-based culture for cultivation with 50 µM and 5 mM Pi supplied. The nutrient solution contained 50 µM/5 mM KH_2_PO_4_. The basal nutrient solution contained 2 mM Ca(NO_3_)_2_, 0.65 mM MgSO_4_, 25 μM FeEDTA, 5 μM MnSO_4_, 50 μM KCl, 2 μM ZnSO_4_, 0.5 μM CuSO_4_, 0.005 μM (NH_4_)_6_Mo_7_O_24_ and 25 mΜ H_3_BO_4_. In addition, K^+^ was supplied in the LP (Low Phosphate condition) solution in the form of 0.95 mM KCl [46]. Three seedlings were transplanted to a sterilized sand-based pot. The sand base contained *Glomus etunicatum* were used for colonization. Tissues (wild type and inoculated plants) were harvested at 40 days post-inoculation. Parts of secondary roots were used for the detection of mycorrhizal colonization, and the rest of the secondary roots were stored at −80 °C after being frozen in liquid nitrogen and were used for subsequent RNA isolation.

### 4.3. Detection of Mycorrhizal Colonization

Maize roots were firstly treated with 10% KOH and heated 1 h at 90 °C, further treated 5 min with 2% HCl solution. Then, the root samples were stained with Trypan Blue and then they were transparent with lactic acid and glycerin. Dyed roots were detected by a microscope (Figure S5).

### 4.4. Total RNA Extraction

Root total RNA was isolated using RNA Plus (Takara, Dalian, China) with the guanidine thiocyanate extraction method. Then, 1.2% agarose gel and the NanoDrop ND-1000 spectrophotometer (Thermo Fisher Scientific, Wilmington, DE, USA) were used to evaluate and quantify RNA, respectively. DNase was used to eliminate the potential trace of genomic DNA in RNA samples.

### 4.5. qRT-PCR

The Roche reverse transcription kit was used to synthesize cDNA first. A 20-µL reaction solution contained 1 µg RNA approximately. Then, synthesized cDNAs were used as templates for qRT-PCR. The qRT-PCR was carried out on Applied Biosystems 7300 using the SYBGREEN kit (Roche, Basel, Switzerland). The qRT-PCR program was used as follows: 95 °C for 10 min, followed by 40 cycles at 95 °C for 15 s and 60 °C for 1 min. The expression level of the maize *Actin 1* gene was used as an internal control. The relative transcript level was calculated as 2^−ΔΔ*C*t^. The relative expression level in the normal plant without tress treatment was normalized to 1. The primer pairs for each of the *ZmPHT1* genes are listed in Supplementary Table S2, and the melting curves are listed in Figure S6.

### 4.6. Yeast Manipulations

The yeast mutant BY4743, which defects *PHO84*, a high-affinity Pi transporter gene [18,47], and the expression vector pYES2 were used in yeast complement experiments. The full length coding sequences of *ZmPHT1s* were amplified from cDNAs using PrimeSTAR Max DNA Polymerase (Takara), which has high amplification efficiency, high sensitivity and high specificity characteristics. The PCR was performed in a 50-μL volume, which contained 25 μL of 2× PrimerSTAR Max Premix, 2 μL diluted cDNA template, 1.25 μL of each specific primer (10 μM of each primer) and 20.5 μL ddH_2_O. The PCR program was used as follows: 35 cycles at 98 °C for 10 s, 56 to 68 °C for 30 s, 72 °C for 1 min and at last, 72 °C for 10 min. Sequencing was used for all PCR products’ verity. Then, PCR productions were subcloned into pYES2 (preserved by our lab). These constructs were transformed into the yeast BY4743. Transformed yeast strains grew in YNB medium to the logarithmic phase, and then were harvested and washed with Pi-free YNB medium. Collected yeast was firstly suspended in Pi-free YNB medium and cultivated until the absorbance at 600 nm was 1.0. Then, 10-fold serial dilution with equal volumes was applied to solid YNB medium (the sample volume was 3 µL) supplied with 20 and 100 µM KH_2_PO_4_ and incubated at 30 °C for 2 days.

## 5. Conclusions

Eight of 13 putative *PHT1* genes were newly identified in the maize elite inbred line B73 genome. All of the 13 genes were complementary verified in the yeast Pi-uptake mutant. The locations of the *ZmPHT1*s on maize chromosomes and duplication events were also investigated in detail. All of the *ZmPHT1* genes were significantly induced under the low Pi supply condition, with the exception of *ZmPHT1;1* (*p* < 0.01). *ZmPHT1;2*, *ZmPHT1;4*, *ZmPHT1;6*, *ZmPHT1;7*, *ZmPHT1;9* and *ZmPHT1;11* were upregulated in arbuscular mycorrhizal. The AM-responsive element (MYCS) and P1BS element can be observed in promoters of the majority of AMF-inducible *ZmPHT1* genes.

## Figures and Tables

**Table 1:** Details of the *PHT1* genes of maize.

Gene Name	Transcript ID	Accession Number	Gene Size (bp)	CDS Size (bp)	Protein Length (AA)	Protein Molecular Mass (Da)	pI	Chromosome
*ZmPHT1;1*	*GRMZM2G326707_T01*	NM_001279426.1	2199	1617	539	58,485.66	7.65	5
*ZmPHT1;2*	*GRMZM2G139639_T01*	NM_001156420.1	1835	1611	537	58,660.21	7.98	7
*ZmPHT1;3*	*GRMZM2G112377_T01*	NM_001112347.2	2175	1629	543	57,899.24	8.11	1
*ZmPHT1;4*	*GRMZM2G170208_T01*	NM_001279982.1	1809	1548	516	57,145.85	9.13	2
*ZmPHT1;5*	*GRMZM2G041595_T01*	NM_001112348.1	1698	1527	509	56,222.71	8.93	7
*ZmPHT1;6*	*GRMZM5G881088_T01*	NM_001112306.1	1971	1662	554	60,579.71	8.15	8
*ZmPHT1;7*	*GRMZM2G075870_T01*	NM_001157730.1	1999	1761	587	63,148.92	8.34	1
*ZmPHT1;8*	*GRMZM2G045473_T01*	NM_001139212.1	1870	1605	535	58,762.9	7.58	2
*ZmPHT1;9*	*GRMZM2G154090_T01*	NM_001112346.1	2020	1623	541	58,752.98	7.63	1
*ZmPHT1;10*	*GRMZM2G159075_T01*	XM_008664847.1	2172	1791	597	65,240.06	9.11	10
*ZmPHT1;11*	*GRMZM2G009779_T01*	NM_001112348.1	1581	1551	517	57,538.36	9.18	2
*ZmPHT1;12*	*GRMZM2G009800_T02*	NM_001112348.1	1846	1770	590	66,071.38	9.41	2
*ZmPHT1;13*	*GRMZM2G070087_T01*	NM_001196972.1	1986	1572	524	57,450.92	8.37	1

CDS, coding sequence; pI, isoelectric point.

**Table 2:** Estimates of the dates for duplication events in maize *PHT1* paralogs.

Paralogous Pairs	Ks	Ka	Ka/Ks	Duplication Rate (Mya)	Duplication Type
ZmPHT1;1/ZmPHT1;9	0.16	0.03	0.19	12.36	Segmental
ZmPHT1;1/ZmPHT1;13	0.43	0.18	0.41	32.72	Segmental
ZmPHT1;8/ZmPHT1;10	0.55	0.36	0.66	42.05	Segmental
ZmPHT1;9/ZmPHT1;13	0.10	0.28	2.76	7.91	Segmental

Mya, Millions of years ago; Ks, synonymous substitutions; Ka, nonsynonymous substitutions; Ka/Ks, nonsynonymous substitutions per synonymous site.

## Supplementary Materials

Supplementary materials can be found at http://www.mdpi.com/1422-0067/17/6/930/s1.

## Abbreviations

AMF	Arbuscular mycorrhizal fungi
qRT-PCR	Quantitative real-time polymerase chain reaction
NJ	Neighbor-joining
Ks	The rate of synonymous substitutions
Ka	The rate of nonsynonymous substitutions
MYCS	Mycorrhizal transcription factor binding sequence
P1BS	PHR1 binding site
