# Supplementary material for: Systematic Identification, Evolution and Expression Analysis of the Zea mays PHT1 Gene Family Reveals Several New Members Involved in Root Colonization by Arbuscular Mycorrhizal Fungi
Source: Int J Mol Sci. 2016 Jun 13;17(6):930. doi: 10.3390/ijms17060930 (PMC4926463; doi:10.3390/ijms17060930)
Supplement: Supplementary file 1 [file ijms-17-00930-s001.pdf]

Supplementary Materials: Systematic Identification, Evolution and Expression Analysis of the *Zea mays* *PHT1* Gene Family Reveals Several New Members Involved in Root Colonization by Arbuscular Mycorrhizal Fungi

Fang Liu, Yunjian Xu, Huanhuan Jiang, Chaosheng Jiang, Yibin Du, Cheng Gong, Wei Wang, Suwen Zhu, Guomin Han and Beijiu Cheng

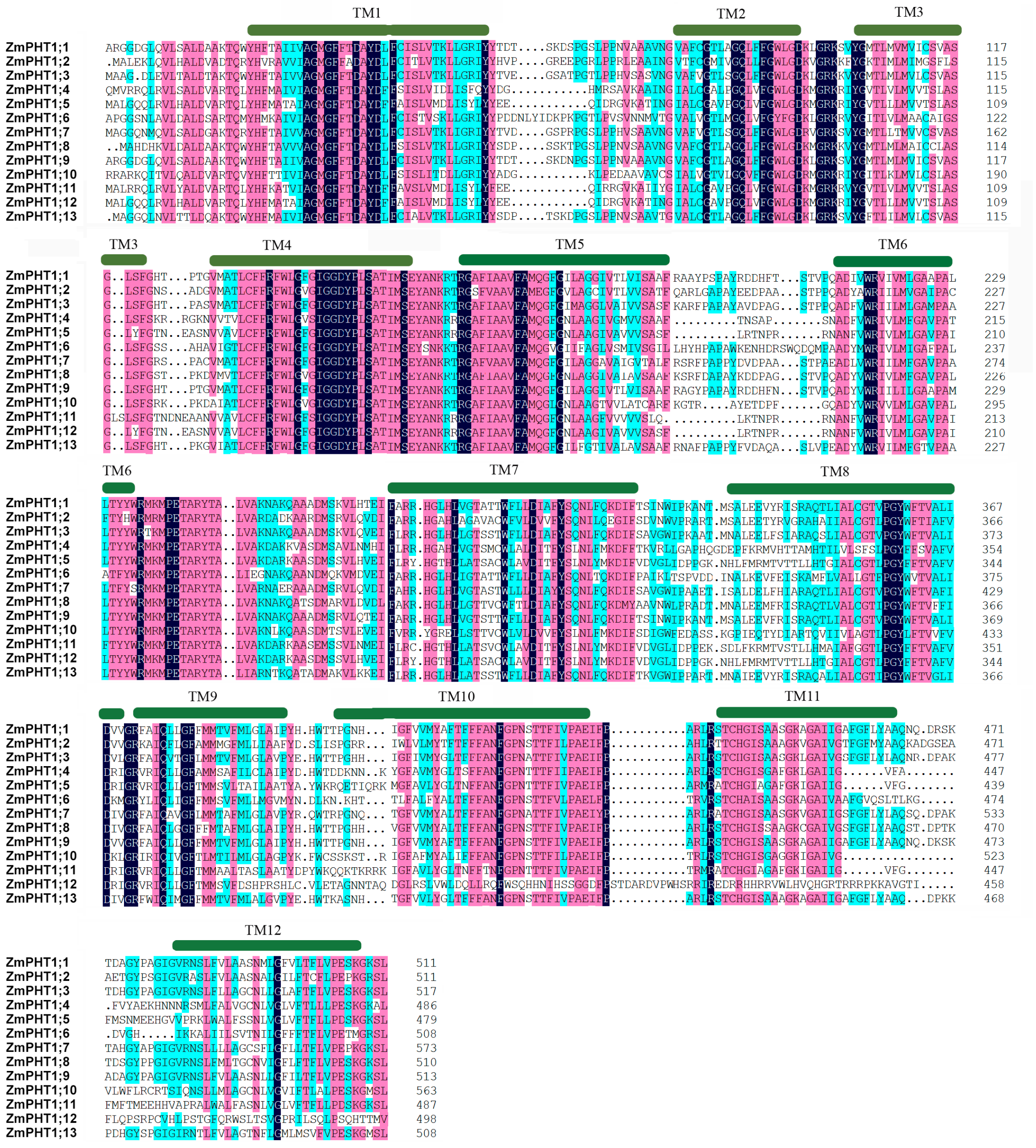

Figure S1. Multiple alignment of maize ZmPt1 to ZmPt13 proteins. Sequence alignment analysis was carried out using multiple alignment of DNAMAN 6.0 program. Identical amino acids are shaded and gaps are indicated by dots. Dark blue, red and light blue shading indicate identical, highly conserved and conserved amino acid residues, respectively. The predicted transmembrane (TM) segments were performed using the programs TMHMM.

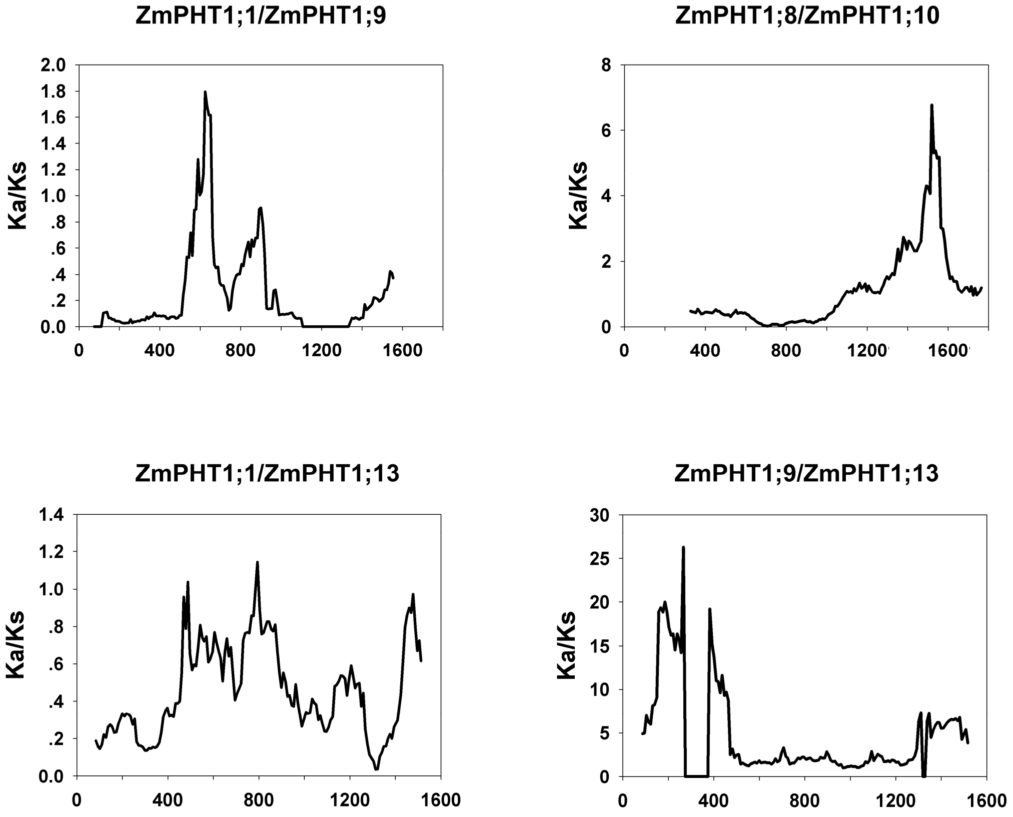

Figure S2. Sliding window plots of representative duplicated *ZmPt* genes in maize. The window size was 150 bp, and the step size was 9 bp.

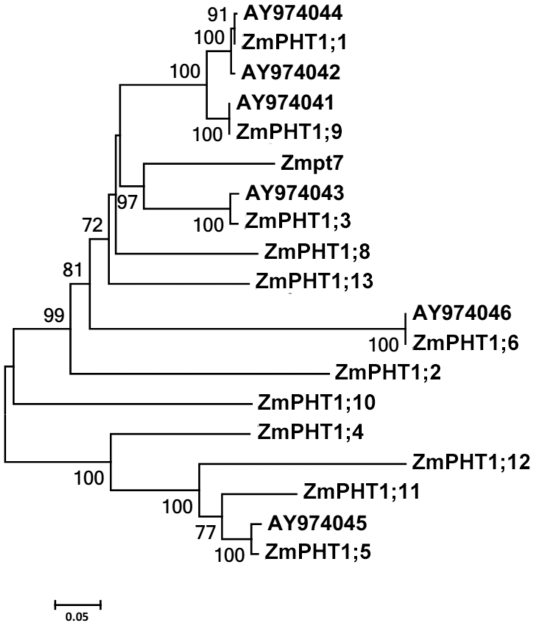

Figure S3. Phylogenetic tree of maize Pht1 proteins from maize inbred line B73 and commercial hybrid line. ZEAma;Pht1;1 (AY974041), ZEAma;Pht1;2 (AY974042), ZEAma;Pht1;3 (AY974043), ZEAma;Pht1;4 (AY974044), ZEAma;Pht1;5 (AY974045), ZEAma;Pht1;6 (AY974046).

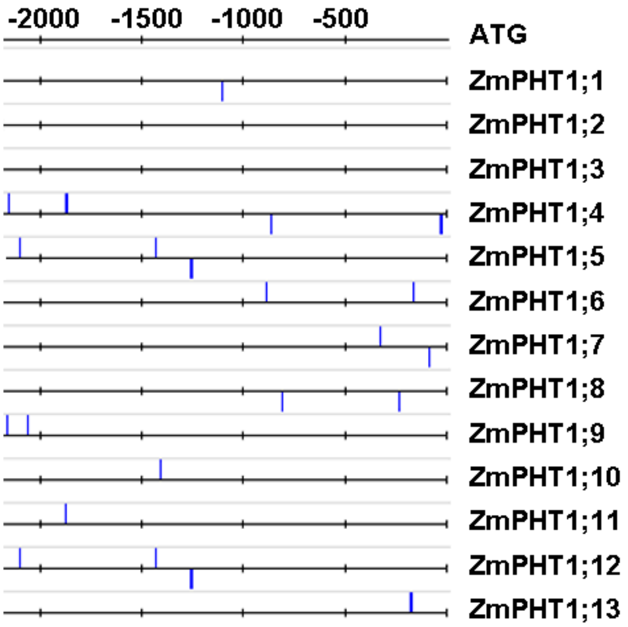

Figure S4. Locations of the CTTC motifs in promoter regions of 13 *ZmPHT1*s. The CTTC motifs are shown in blue and are located 2200 bp upstream of the start codon ATG.

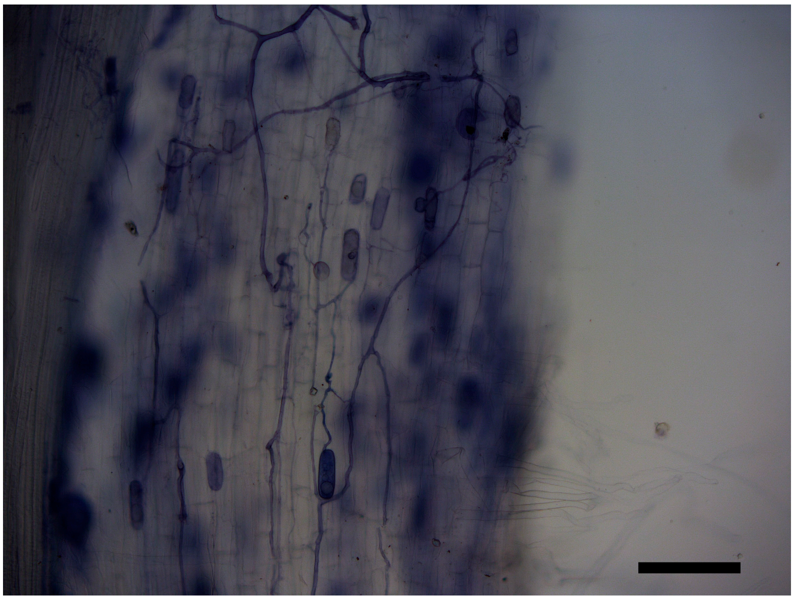

Figure S5. Trypan blue staining of colonized maize roots, 40 day. Maize plants were inoculated with *Glomus etunicatum*. Scale bar is 100 μm.

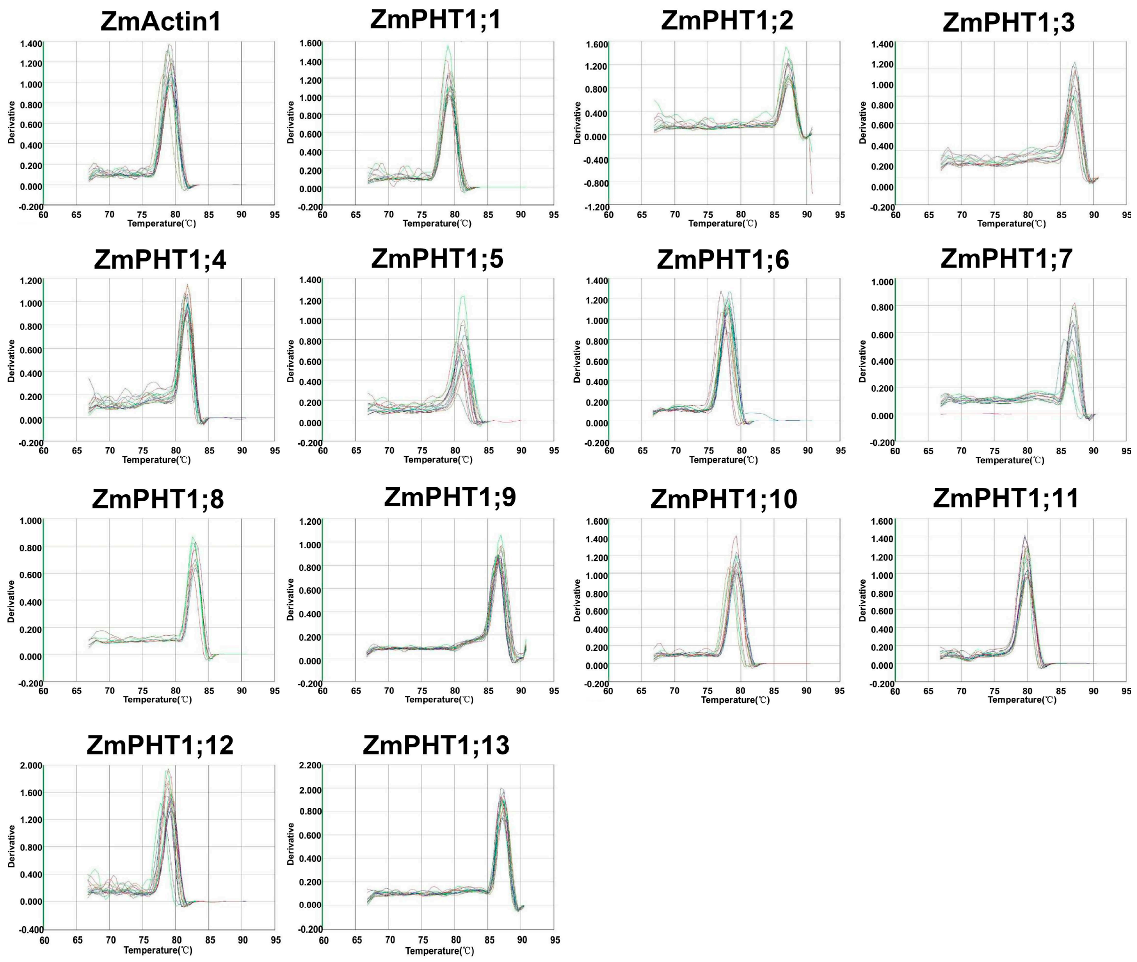

Figure S6. Melting curves for each of *ZmPHT1* genes qRT-PCR primer.

Table S1. Motifs and amino acid sequences of ZmPt proteins.

Motif	Sequence
motif 1	TLCFFRFWLGFGIGGDYPLSATIMSEYANKRTRGAFIAAVFAMQGFGILA
motif 2	ADYVWRIVLMFGAVPALLTYYWRMKMPETARYTALVAKNAKQATSDMARV
motif 3	GLQVLSALDAAKTQWYHFTAIIVAGMGFFTDAYDLFCISLVTKLLGRIYY
motif 4	MYAFTFFFANFGPNSTTFIVPAEIFPARLRSTCHGISAASGKAGAIIGAF
motif 5	AAVNGVAFCGTLAGQLFFGWLGDKLGRKSVYGMTLMLMVICSVASGLSFG
motif 6	FGLFSREFARRHGLHLVGTSTTWFLLDIAFYSQNLFQKDIFTSINWIPKA
motif 7	QTLIALCGTVPGYWFTVALIDVVGRFAIQLLGFFMMTVFMLGLAIPYHHW
motif 8	YPAGIGVRNSLFVLAASNMLGFVLTFLVPESKGKSLEEMSGEAEDSEEEP
motif 9	VAIVVSASFKARFPAPAYAVDPAGSTPPQ
motif 10	NTMSALEEVYRISRA

Table S2. qRT-PCR primers.

Primer Names	Primer Sequence
Zmpt1F	GCCTTCACCTTCTTCTTCGC
Zmpt1R	CCGTCTTGCTCCTGTCCTG
Zmpt2F	GTTCGGGTTCATGTATGCG
Zmpt2R	GCAGGAAGCAGGTGAAGAGTA
Zmpt3F	TCGTCGGCTCCTTCGGGTTC
Zmpt3R	ACACGGGCACTGTGCGGTTG
Zmpt4F	TGCCTCGCTATCCCTTAT
Zmpt4R	GCTTCGGTTGTTGTTATGTT
Zmpt5F	CGCAGTCTTGTATGGCTTGA
Zmpt5R	GCGCCTATCTTCCCGAAT
Zmpt6F	GGACACCTGCCTTACATTGCC
Zmpt6R	TCGTAGGCGTCGGTGAAGAA
Zmpt7F	GCCTCCTCCGCATCCACTA
Zmpt7R	CGTCGGTGAAGAAGCCCATC
Zmpt8F	CACTTTCTTCTTCGCCAACTT
Zmpt8R	ACAGGAACCCAAATGACCC
Zmpt9F	ACCACATCGGCTTCGTCG
Zmpt9R	CCGCCTTGCTCTTGTCCTG
Zmpt10F	TCTGGTTCCTCCGCTGTC
Zmpt10R	AAGTTCCTCGTTCCGTTG
Zmpt11F	ATCATCGGCGTGTTTGGC
Zmpt11R	CAGCGACTTACCTTTGGAAT
Zmpt12F	TCCCAACACCACAACATTC
Zmpt12R	TCTTCCAGCGACTTACCTT
Zmpt13F	CGTCCTCTACGGGCTCACCT
Zmpt13R	TGTTGCGGATGCCGATGC
ZmActinF	GGGATTGCCGATCGTATGAG
ZmActinR	GAGCCACCGATCCAGACACT

Table S3. Gene name and Gene ID of Pht1 genes in rice, sorghum and *Brachypodium*.

Gene Name	Gene ID
BdPt1	Bradi1g00700.1
BdPt2	Bradi1g42610.1
BdPt3	Bradi1g52590.1
BdPt4	Bradi1g75020.1
BdPt5	Bradi1g75030.1
BdPt6	Bradi1g76010.1
BdPt7	Bradi2g45520.1
BdPt8	Bradi3g12590.1
BdPt9	Bradi3g27680.1
BdPt10	Bradi5g02730.1
BdPt11	Bradi5g02750.1
BdPt12	Bradi5g02760.1
BdPt13	Bradi5g02770.1
OsPt1	LOC_Os03g05620
OsPt2	LOC_Os03g05640
OsPt3	LOC_Osl0g30770
OsPt4	LOC_Os04gl0750
OsPt5	LOC_Os04gl0690
OsPt6	LOC_Os08g45000
OsPt7	LOC_Os03g04360
OsPt8	LOC_Osl0g30790
OsPt9	LOC_Os06g21920
OsPt10	LOC_Os06g21950
OsPt11	LOC_Os01g46860
OsPt12	LOC_Os03g05610
OsPt13	LOC_Os04g10800
SbPt1	Sb01g046890.1
SbPt2	Sb06g002800.1
SbPt3	Sb01g047910.1
SbPt4	Sb01g020580.1
SbPt5	Sb01g046900.1
SbPt6	Sb07g023780.1
SbPt7	Sb01g020570.1
SbPt8	Sb02g009880.1
SbPt9	Sb06g002560.1
SbPt10	Sb06g002540.1
SbPt11	Sb03g029970.1

Table S4. Comparisons of *Pht1* genes in maize hybrid and inbred lines.

Gene Name	Gene Name	Protein Identity
ZEAma;Pht1;1 (AY974041)	*ZmPt9*	100%
ZEAma;Pht1;2 (AY974042)	*ZmPt1*	98.17%
ZEAma;Pht1;3 (AY974043)	*ZmPt3*	97.62%
ZEAma;Pht1;4 (AY974044)	*ZmPt1*	99.81%
ZEAma;Pht1;5 (AY974045)	*ZmPt5*	98.23%
ZEAma;Pht1;6 (AY974046)	*ZmPt6*	100%
